# A Mathematical Model of Comprehensive Test-and-Treat Services and HIV Incidence among Men Who Have Sex with Men in the United States

**DOI:** 10.1371/journal.pone.0029098

**Published:** 2012-02-10

**Authors:** Stephen W. Sorensen, Stephanie L. Sansom, John T. Brooks, Gary Marks, Elizabeth M. Begier, Kate Buchacz, Elizabeth A. DiNenno, Jonathan H. Mermin, Peter H. Kilmarx

**Affiliations:** 1 Division of HIV/AIDS Prevention, National Center for HIV/AIDS, Viral Hepatitis, STD, and TB Prevention, Centers for Disease Control and Prevention, Atlanta, Georgia, United States of America; 2 New York City Department of Health and Mental Hygiene, New York, New York, United States of America; INSERM & Universite Pierre et Marie Curie, France

## Abstract

**Background:**

Early diagnosis and treatment of HIV infection and suppression of viral load are potentially powerful interventions for reducing HIV incidence. A test-and-treat strategy may have long-term effects on the epidemic among urban men who have sex with men (MSM) in the United States and may achieve the 5-year goals of the 2010 National AIDS Strategy that include: 1) lowering to 25% the annual number of new infections, 2) reducing by 30% the HIV transmission rate, 3) increasing to 90% the proportion of persons living with HIV infection who know their HIV status, 4) increasing to 85% the proportion of newly diagnosed patients linked to clinical care, and 5) increasing by 20% the proportion of HIV-infected MSM with an undetectable HIV RNA viral load.

**Methods and Findings:**

We constructed a dynamic compartmental model among MSM in an urban population (based on New York City) that projects new HIV infections over time. We compared the cumulative number of HIV infections in 20 years, assuming current annual testing rate and treatment practices, with new infections after improvements in the annual HIV testing rate, notification of test results, linkage to care, initiation of antiretroviral therapy (ART) and viral load suppression. We also assessed whether five of the national HIV prevention goals could be met by the year 2015. Over a 20-year period, improvements in test-and-treat practice decreased the cumulative number of new infections by a predicted 39.3% to 69.1% in the urban population based on New York City. Institution of intermediate improvements in services would be predicted to meet at least four of the five goals of the National HIV/AIDS Strategy by the 2015 target.

**Conclusions:**

Improving the five components of a test-and-treat strategy could substantially reduce HIV incidence among urban MSM, and meet most of the five goals of the National HIV/AIDS Strategy.

## Introduction

Men who have sex with men (MSM) represent about 2% of the U.S. population [1] but accounted for 57% of new HIV diagnoses in 2009. In addition, MSM with a history of injection drug use accounted for another 3% of new diagnoses [2]. Not only are MSM the population most severely affected by HIV, they are the only risk group in which new HIV infections have been increasing steadily since the early 1990s [3]. A 2008 surveillance project employing venue-based sampling found that one in five (19%) MSM in 21 major U.S. cities were infected with HIV [4]. In addition, nearly half (44%) were unaware of their infection and 55% had not been tested for HIV infection in the previous 12 months.

Several modeling studies have shown that a test-and-treat approach to HIV infection, whereby at-risk individuals are tested frequently and linked to early treatment if diagnosed, could reduce HIV epidemics [5]. One dynamic transmission model of males and females suggested that a strategy of universal screening with immediate initiation of effective antiretroviral therapy could virtually eliminate the HIV epidemic in South Africa within 50 years [6]. Results from a similar dynamic transmission model of MSM and injection drug users indicated that expanding access to antiretroviral treatment, including earlier initiation of antiretroviral therapy (ART) (i.e., at a CD4 count of 350 versus 200 cells/mm^3^) could substantially reduce the HIV epidemic in British Columbia, Canada [7]. A dynamic model based on San Francisco MSM also showed improvement from earlier initiation of ART therapy [8].

However, an individual simulation model of test-and-treat for males and females in Washington, D.C., showed a modest impact on HIV transmission over the next 5 years [9]. A dynamic transmission model that explored the implications for low- and high-risk U.S. populations of males and females also showed more modest levels of improvement in HIV incidence over time [10]. These North American models [7]–[10] did not include the entire paradigm of test-and-treat interventions or discuss their interactions.

We used a dynamic mathematical model to estimate the potential benefit of a proposed test-and-treat strategy to reduce new HIV infections among MSM in an urban population. We evaluated, singly and in combination, a set of five interventions that could comprise a test-and-treat strategy: routine repeated HIV screening, notification of results, linkage of HIV-infected persons to care, initiation of treatment, and HIV viral load suppression, defined as undetectable viral load based on the limits of detection. We estimated the effect of these interventions as currently practiced on the incidence of new HIV infections, and then explored the same effects if these interventions were implemented with intermediate, potentially feasible, levels of improvement and then with maximum (best-case) levels of improvement. We included these idealistic, best-case scenarios to indicate the maximum number of new HIV cases that could be prevented over time and to compare the extent to which more feasible programmatic goals might achieve comparable results.

The 2010 U.S. National HIV/AIDS Strategy sets goals for addressing the HIV epidemic by 2015 that include: 1) lowering by 25% the annual number of new infections, 2) reducing by 30% the HIV transmission rate, 3) increasing to 90% the proportion of persons living with HIV infection who know their HIV status, 4) increasing to 85% the proportion of newly diagnosed patients linked to clinical care, and 5) increasing by 20% the proportion of HIV-infected gay and bisexual men with an undetectable HIV RNA viral load [11]. We assessed the ability of our five-part test-and-treat strategy – using values representing both intermediate and best-case improvements in current practice – to reduce the HIV epidemic in urban MSM over 20 years and to achieve national HIV goals by 2015.

## Methods

### Dynamic compartment model

We constructed a dynamic compartmental model of HIV infections among an urban population of MSM (based on New York City). Our model included only sexual transmission of HIV infection between MSM; it did not include MSM who are also injection drug users.

Our model's parameters for behavior, incidence, and treatment were derived from data in CDC's National HIV Behavioral Surveillance System (NHBS), the New York City HIV/AIDS Reporting System (HARS), and the CDC-sponsored HIV Outpatient Study (HOPS). The New York City HARS is a state-required registry. The NHBS and HOPS obtained written, informed consent from participants. For NHBS the participants were anonymous. The data we analyzed were extracted from original datasets. Data extracts from all sources were stripped of any elements that could identify individuals. We explained our purpose in requesting the data to the data managers, but were not required to obtain ethics approval from an ethics committee or review board.

NHBS is used to monitor prevalence and trends in HIV-related risk behaviors, HIV testing, and use of HIV prevention services among populations at high risk for acquiring HIV. In 2008, NHBS among MSM (NHBS-MSM2) collected venue-based sampling data from 21 metropolitan statistical areas (MSAs) using an anonymous cross-sectional interview of men at venues where MSM congregate, such as bars, clubs, and social organizations. Respondents gave written, informed consent for the interview and were offered anonymous HIV testing, regardless of self-reported HIV infection status [4]. The New York City HARS is a population-based registry required by New York State that collects dates of HIV diagnosis and initial and subsequent CD4 cell counts for New York City residents who are diagnosed with HIV infection [12]. The HOPS is a prospective longitudinal cohort study of HIV-infected adults in care, many of whom are receiving antiretroviral treatment, seen at urban HIV specialty clinics in the United States that participated in the study. The study protocol is approved annually by each participating clinic's institutional review board. All study participants provide written, informed consent. The HOPS includes data on HIV RNA viral load, CD4 cell count, and treatment regimens over time for each patient [13], [14].

We divided the MSM population into compartments according to five infection states, five age groups, and two sexual activity levels based on the number of partners an individual had in a year. The technical appendix (Appendix S1) contains more complete details regarding our model and methods. Table 1 lists key parameters used in the model, their values, and their sources. The modeled infection states included HIV-uninfected MSM, who are assumed to become HIV-infected through anal intercourse based on the likelihood of forming a partnership with an infected person and the probability of HIV transmission within that partnership. Once infected, an individual was assumed to progress through the stages of acute infection, early latent infection, latent infection, late infection, and AIDS. At each infection state, HIV-infected individuals may become aware of their infection through testing, and linked to care. HIV-infected individuals in care received or did not receive ART, with probability of ART initiation at a given level of CD4 cell count derived from estimates in the HOPS. The infection states corresponded to CD4 cell counts: early latent infection to a CD4 count greater than 500 cells/mm^3^, latent infection to a CD4 count between 350 and 500 cells/mm^3^, late infection to a CD4 count between 200 and 350 cells/mm^3^, and AIDS to a CD4 count less than 200 cells/mm^3^.

**Table 1:** Values for input parameters for the model and references.

Description Of Parameter	Value	Range
Epidemiological parameters		
Condom efficacy, [27]–[30]	85%	
Reduction in HIV transmission among persons with suppressed viral load compared with unsuppressed, [40]	90%	80%–99%
Percent of HIV+ who know their status: Age 18–24, [4]	0.319	
Percent of HIV+ who know their status: Age 25–34, [4]	0.451	
Percent of HIV+ who know their status: Age 35–44, [4]	0.626	
Percent of HIV+ who know their status: Age 45–54, [4]	0.741	
Percent of HIV+ who know their status: Age 55–64, [4]	0.763	
Fraction unwilling to be tested until AIDS, [4]	5%	
Behavioral parameters		
Percent of the time using condoms for unaware, [22]–[26]	50%	
Percent of the time using condoms for aware, [22]–[26]	75%	
Fraction serosorting among aware, [21]	15%	
Unprotected MSM per-contact transmission probability by disease state of HIV+ partner		
Acute, HIV-uninfected receptive*	0.0560	
Early Latent and Latent (Asymptomatic), HIV-uninfected receptive*	0.0048	
Late (Symptomatic), HIV-uninfected receptive*	0.0096	
AIDS, HIV-uninfected receptive*	0.0294	
Acute, HIV-uninfected insertive*	0.0123	
Early Latent and Latent (Asymptomatic), HIV-uninfected insertive*	0.0011	
Late (Symptomatic), HIV-uninfected insertive*	0.0021	
AIDS, HIV-uninfected insertive*	0.0065	
Population parameters		
Population of sexually active MSM in 2004**	194,000	
Test-and-treat Interventions		
Annual testing rate	24%	48%–95%
Notification rate for conventional testing	80%	90%–100%
Percent linked to care in 12 months	70%	85%–100%
Retention in care, [39]	85%	100%
Percent achieving viral load suppression	80%	90%–98%
Initiate treatment with ART	CD4 300 cells/mm^3^	CD4 500 cells/mm^3^ or at diagnosis

*Calculated in Appendix S1.

**Calculated from New York City Community Health Survey.

We used per-contact transmission probabilities from a study of protected and unprotected, insertive and receptive anal intercourse among MSM [15]. We modified those transmission probabilities to obtain per-contact transmission probabilities by each stage of infection using estimates in an observational study of treatment-naïve couples engaged in heterosexual intercourse [16]. (Appendix S1.) Other studies have shown that transmission rates from anal sex are similar overall for heterosexual and MSM groups, but MSM transmission rates by disease state have not been published [17]. We estimated the rate of transition from one stage of infection to another from an observational study [16].

Individuals in the various age groups (i.e., 18–24, 25–34, 35–44, 45–54, and 55–64 years) entered the sexually active population each year by aging, initiating sex with other men or immigrating into the New York City geographic area. Individuals left the modeled population due to older age, HIV/AIDS-related death, or death from non-HIV/AIDS causes.

For the period before 2009, we used published guidelines to inform our assumption about the timing of initiation of ART. Initiation was generally recommended at CD4<350 cells/mm^3^. Since December 2009, treatment generally has been recommended at CD4<500 cells/mm^3^ and was recommended or considered optional at CD4>500 cells/mm^3^ [18]. However, for 2009 and beyond, we estimated the current practice average CD4 count for initiation of treatment using data from the HIV Outpatient Study. Survival estimates for individuals receiving ART depended on age and CD4 cell count at ART initiation [19], [20].

We validated our model against epidemic data from the Department of Mental Health and Hygiene in New York City. First, we determined both a constant annual input for each age group of uninfected MSM and the rate that persons exited from sexual activity by calibrating our estimates to each age group's reported population in 2004. Then we fit a least squares curve to reported diagnoses in AIDS from 1996–2008 based on surveillance data from New York City. The percentage of the population by age and activity already infected with HIV at the start of the simulation in 1975 was selected to provide the best least squares fit.

The effects of HIV testing and awareness of HIV-positive status on reducing secondary HIV transmission were based in part on our assumption that diagnosed individuals decrease risky behaviors that could transmit HIV infection to their sexual partners. In our model, persons who were diagnosed with HIV infection decreased their number of sexual acts with HIV-uninfected partners by 15% but kept the total number of sexual acts the same [21]. They increased their use of condoms for anal sex from 50% to 75% [22]–[26]. We assumed a condom efficacy of 85% per sexual act [27]–[31] and varied assumptions about condom use in sensitivity analyses.

The prevalence of male circumcision is important for an MSM model in the United States. We estimated prevalence at 60% [32]. Circumcision reduces the insertive infectivity for uninfected MSM by 60%, based on results from three randomized controlled trials in Africa on the efficacy of circumcision in preventing female-to-male HIV transmission [33]–[35].

### Interventions for a test-and-treat strategy

We evaluated the effect on the MSM HIV epidemic of a set of five interventions that could be included in a future test-and-treat strategy, both individually and combined. Those interventions included an increased annual HIV testing rate, improved notification of test results, improved linkage to care, earlier initiation of ART and more complete achievement of HIV viral load suppression. We established a current practice estimate for each intervention based on published data and considered both intermediate and best-case levels of improvement in current practice. The best-case levels of improvement were very optimistic while the intermediate levels of improvement were roughly mid-way between current and best-case levels and may be more feasible in practice. Parameters used for each intervention included in our test-and-treat strategies are listed in Table 1.

### Annual rates of testing for HIV infection

We estimated an annual testing rate that produced the annual number of newly diagnosed MSM with AIDS (CD4 count less than 200 cells/mm^3^) in New York City in 1997–2009 [36] and produced the estimated percent of infected individuals (by age group) aware of their infection in 2008 [4]. The estimated overall annual testing rate was 24% per year for persons who were HIV-infected, unaware of their infection, and willing to be tested; but the rate varied by age and infection state – our model assumed persons not HIV-infected were tested at the same rate. HIV-infected individuals who were older or who were at a more advanced stage of HIV disease were more likely to be tested and diagnosed than were younger persons or persons with less advanced HIV disease. We examined the benefits of increasing the annual testing rate to an intermediately effective value of 48% and a best-case effective value of 95%. In all analyses we assumed that 5% of HIV-infected MSM would not be tested until they had a CD4 count less than 200 cells/mm^3^ [4].

### Notification of test results

An individual who was tested must be notified of the result and linked to care to benefit from a test-and-treat strategy. We assumed that each year half of the tested persons were tested using rapid point-of-care tests and that all of those newly diagnosed with HIV infection received their results. We assumed that the remaining half were tested with conventional tests and that 80% of those newly diagnosed with HIV received their results [37], [38]. We examined the benefit of improving notification of a positive result following a conventional test to 90% and 100%, in both the intermediate and best cases, respectively.

### Linkage to care and retention in care

We estimated 70% of MSM who tested positive were linked to care in the first year [39]. Diagnosed persons not linked to care in the first year were given a 10% annual probability of linkage in each subsequent year until they reached AIDS, or a CD4 count of 200 cells/mm^3^, at which time they had 100% probability of linkage to care. We assumed an annual 15% drop-out-of-care rate among individuals who had been linked to care but had not initiated ART [40]. We assumed an annual 10% reentry-to-care rate among individuals who had been linked to care but had dropped out before ART initiation. Once their CD4 count declined to 200 cells/mm^3^, all individuals were assumed to be relinked. We considered 100% retention in care in a sensitivity analysis.

We examined the benefits of improving linkage to care in the first year to an intermediate value of 85% (consistent with the National HIV/AIDS Strategy 2015 target) and a best-case value of 100%. Persons had to be in care to initiate ART.

### Initiation of ART

We estimated current practice for ART initiation based on CD4 cell count at initiation in the HIV Outpatient Study. For 2004–2009, the median and mean CD4 counts at ART initiation were 300.0 cells/mm^3^ and 299.9 cells/mm^3^ respectively for 381 MSM who had a CD4 cell count available. Of those 381 MSM initiating ART, 29.1% had CD4 counts less than 200 cells/mm^3^, 34.6% had CD4 counts between 200 and 349 cells/mm^3^, 23.4% had CD4 counts between 350 and 499 cells/mm^3^, and 12.9% had CD4 counts of 500 cells/mm^3^ or greater. We examined the effect of starting ART at 500 cells/mm^3^ as an intermediate case, and at diagnosis (regardless of CD4 cell count level) as a best case. Persons already in care but not receiving ART also started ART at these new levels.

### Viral load suppression

We estimated that 80% of persons who started ART achieved viral load suppression, defined as undetectable viral load based on the limit of detection. This estimate took into account retention in care, adherence to treatment and the immune response to treatment [41]. We examined 90% viral load suppression in the intermediate case and 98% in the best case[42].

We further assumed that persons who achieved viral load suppression experienced a 90% decline in the rate of per-contact HIV transmission compared with persons who do not achieve viral load suppression [43], [44]. In sensitivity analysis, we varied that assumption from an 80% to a 99% decline in the rate of per-contact transmission.

### Analysis plan

We simulated annual testing rates, notification of results, linkage to care, initiation of treatment and viral load suppression for the time frame 1996 to 2009 to project future trends based on our current practice. We projected trends from 2010 through 2029 and applied our set of test-and-treat interventions to current practice improved to both intermediate and best-case levels. We also examined the projected number of new HIV infections and other model outcomes for the year 2015, comparing current with improved practices, to determine if our proposed test-and-treat strategy could meet the National HIV/AIDS Strategy goals for urban MSM. We estimated the impact of each intervention separately and of all interventions combined.

We performed sensitivity analyses that considered risk compensation to determine the consequences if individuals receiving ART (regardless of HIV RNA viral load) decided not to continue using condoms with the same frequency [45]. We determined the threshold value for the decline in condom use among all infected and uninfected MSM that would eliminate all benefits of our proposed test-and-treat strategy, assuming only intermediate levels of improvement to the individual interventions. We considered the “high risk” scenario where individuals on ART stopped using condoms [46].

Although retention in care is not explicitly part of the National HIV/AIDS Strategy goals, we considered the effect of improving retention in care among persons not receiving ART from 85% to 100%.

## Results

### Reduction in new infections using proposed test-and-treat strategy

**Figure 1:**
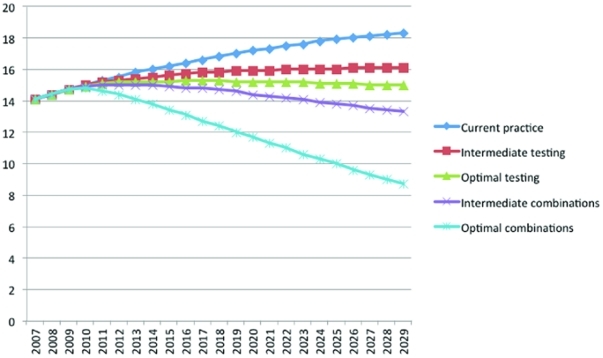
Prevalence of HIV infections over 20 years for MSM in New York City, percent.

Under our estimated current practice for annual HIV testing, notification of results, linkage to care, initiation of ART and viral load suppression, our model projected that 53,178 cumulative new HIV infections would occur in this population of MSM over the next 20 years (Table 2). This projection was associated with an increase in prevalence from 15.0% in 2010 to 18.3% in 2029 (Figure 1). Our prevalence estimate falls between that of 13.7% (95% C.I. 6.0%–28.3%) based on 55 MSM from the New York City Health and Nutrition Examination Survey 2004 [47] and 29% (95% C.I. 25%–33%) based on 462 MSM in New York City in NHBS-MSM2 2008 [4].

**Table 2:** Reduction in new infections from HIV annual testing, notification, linkage to care, treatment, and viral load suppression.

Description	Intermediately Effective Value for Parameter	Total New Infections Over 20 Years	% Decrease Current Practice	Best-case Effective Value for Parameter	Total New Infections Over 20 Years	% Decrease from Current Practice
Current practice		53,178			53,178	
Increase annual testing rate, Current = 24%	48%	45,035	15.3%	95%	41,085	22.7%
Increase notification, Current = 80%	90%	52,686	0.9%	100%	52,230	1.8%
Increase linkage, Current = 70%	85%	52,077	2.1%	100%	50,991	4.1%
ART Initiation Current = CD4 count of 300 cells/mm^3^	CD4 count = 500 cells/mm^3^	48,674	8.5%	At diagnosis	46,943	11.7%
Increased viral load suppression, Current = 80%	90%	47,453	10.8%	98%	42,521	20.0%
Combination of all interventions		32,284	39.3%		16,411	69.1%

When all interventions were simultaneously implemented at intermediate levels of improvement (including beginning ART at a CD4 count of 500 cells/mm^3^), compared with the base case, the projected cumulative number of new HIV infections over 20 years was 32,284, a 39.3% reduction. Among the individual interventions, those with the largest effect on reducing cumulative new infections over 20 years were increasing the annual testing rate among HIV-infected MSM from 24% to 48% (associated with 15.3% fewer infections), increasing viral load suppression among persons receiving ART from 80% to 90% (associated with 10.8% fewer infections) and initiating ART at a CD4 count of 500 cells/mm^3^ (associated with 8.5% fewer infections) (Table 2).

**Figure 2:**
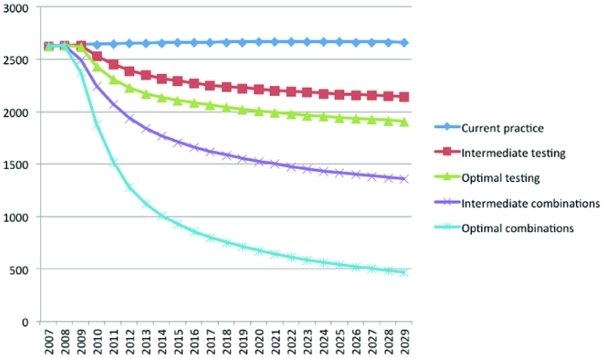
Annual number of new HIV infections over 20 years for MSM in New York City.

Under intermediate levels of improvement for all interventions, the prevalence of HIV infections in 2029 was reduced from 18.3% to 13.3%. (Figure 1), and the annual number of new HIV infections in 2029 was reduced from 2,661 under base case estimates to 1,355 (a 49.1% reduction) (Figure 2).

When all five interventions were simultaneously implemented at best-case levels of improvement (including beginning ART at HIV diagnosis), compared with the base case, the cumulative number of new infections over 20 years was 16,411, a 69.1% reduction. For single interventions each implemented to best-case levels of improvement, reductions in new infections over 20 years ranged from 22.7%, for an increase in the annual testing rate, to 1.8%, for increased notification (Table 2). The prevalence of HIV infections in 2029 was reduced to 8.7% (Figure 1), and the annual number of new HIV infections in 2029 was reduced to 467 (an 82.5% reduction) (Figure 2).

### National HIV/AIDS Strategy 2015 goals

Implementing our proposed set of test-and-treat interventions to intermediate levels of improvement, including initiation of ART at CD4 counts less than 500 cells/mm^3^, would meet four of the five National HIV/AIDS Strategy goals by 2015. The only unmet goal was that of increasing to 90% the proportion of MSM living with HIV who would know their status, due largely to the 5% of men who are not tested until they progress to AIDS. With our combination of interventions implemented to their best-case levels of improvement, all five of the goals were met by 2015 (Table 3).

**Table 3:** Test-and-treat for MSM in New York City and the National HIV/AIDS Strategy for the United States, 2015.

National 2015 goals/Results for each intervention)	Annual Number of New Infections(↓25%)	HIV Transmission Rate(↓30%)	% MSM Living with HIV and Aware(90%)	% Newly Diagnosed Linked to Care in 1 Year(85%)	% Diagnosed with Undetectable Viral Load(↑20%)
Current practice	2,657	8.56%	65.3%	70.0%	51.7%
**Goal based on current practice**	**1,993**	**5.99%**	**90.0%**	**85.0%**	**62.0%**
Increase annual testing rate from 24% to 48%	2,290	7.64%	81.0%	70.0%	46.6%
Increase conventional testing notification from 80% to 90%	2,635	8.50%	66.1%	70.0%	51.5%
Increase linkage from 70% to 85%	2,616	8.41%	65.7%	**85.0%**	53.3%
ART at CD4 count = 500 cells/mm^3^	2,461	7.90%	67.1%	70.0%	59.1%
Increase viral load suppression from 80% to 90%	2,407	8.03%	66.6%	70.0%	58.8%
All intermediately effective, including ART at CD4 count = 500 cells/mm^3^	**1,707**	**5.93%**	84.5%	**85.0%**	**63.2%**
All best-case effective, including ART at diagnosis	**923**	**3.56%**	**93.6%**	**100%**	**86.3%**

Figures in boldface indicate the intervention or combination of interventions that achieve each National HIV/AIDS Strategy goal.

### Sensitivity analyses

When we modeled implementation of intermediate levels of improvement in each of the test-and-treat interventions, but individuals receiving ART used condoms 50% of the time instead of 75%, a 31.6% reduction in new HIV cases occurred over 20 years, compared with the 39.3% reduction we observed without this proposed risk compensation. (Table 4) In the “high risk” case, where individuals receiving ART stopped using condoms, there was a 17.1% reduction in the number of new HIV infections.

**Table 4:** Sensitivity analyses for MSM in New York City.

	Intermediate levels of improvement to all interventions		Best-case levels of improvement to all interventions	
Scenarios	# of new infections over 20 years	% change from current practice	# of new infections over 20 years	% change from current practice
Current practice*	53,178		53,178	
Combination of all interventions	32,284	39.3%	16.411	69.1%
50% condom use by persons receiving ART	36,366	31.6%	20,861	60.8%
0% condom use by persons receiving ART	44,091	17.1%	29,963	43.3%
50% condom use by everyone	53,053	2.4%	30,114	43.4%
80% reduction in per-contact transmission under viral load suppression	39,166	26.4%	24,063	54.8%
99% reduction in per-contact transmission under viral load suppression	25,748	51.6%	9,862	81.5%
100% retention in care	29,187	45.1%	14,709	72.3%

*The sensitivity analyses' results are compared to the current practice in which HIV-infected MSM aware of their infection use condoms for 75% of sex acts, compared with condom use for 50% of sex acts among persons who are HIV-infected but unaware of it. In addition, among HIV-infected MSM who achieve viral load suppression receiving ART in the current practice analysis, per-contact transmission risk is reduced 90% compared to HIV-infected MSM whose viral load is not suppressed.

Threshold analysis indicated that a decrease in condom use to 50% by all MSM (HIV-infected and not HIV-infected, receiving ART and not receiving ART) would have eliminated nearly all of the reduction in new infections associated with having implemented our combination of test-and-treat interventions to intermediate levels of improvement(Table 4). 

When each of the interventions in our proposed test-and-treat strategy were implemented to intermediate levels of improvement, but the reduction in the per-contact transmission rate risk was 80% instead of 90% among MSM with viral load suppression, then the reduction in new cases over 20 years was 26.4% (Table 4) compared with 39.3% (Table 2). When the reduction in per-contact transmission rate was 99% instead of 90%, reduction in new infections was 51.6%.

When annual retention in care was increased from 85% to 100%, the reduction in cumulative new HIV infections over 20 years with intermediate and best-case levels of improvement in each intervention in the proposed test-and-treat strategy were 45.1% (from 39.3%) and 72.3% (from 69.1%), respectively (Table 4).

## Discussion

Using a dynamic, compartmental model our analysis indicated that a five-component test-and-treat strategy such as we have proposed might dramatically reduce new HIV infections over the next 20 years within a heavily affected U.S. population, namely urban MSM. Our strategy required implementing multiple interventions to ensure widespread and frequent testing of the at-risk populations and greater and more comprehensive provision of treatment. Our analysis explored the effect of implementing each intervention individually and combined to both intermediate and best-case levels of improvement.

While improving each intervention individually had some effect, the most significant impact resulted from improving all simultaneously. Even with intermediate levels of improvement in the implementation of these interventions, their combination reduced the cumulative number of new HIV infections over 20 years by 39.3% and reduced HIV prevalence from a projected 18.3% to 13.3%.

Among individual interventions, the most effective was increasing the annual testing rate. Diagnosing previously undiagnosed HIV-infected individuals was associated with a substantial reduction in risk behavior and enabled infected individuals to enter care with the possibility of achieving viral load suppression. However, attaining a 48% annual testing rate will likely require considerably expanded HIV screening and potentially costly outreach to the infected, undiagnosed.

Our findings suggest that initiating ART at the intermediate CD4 count of 500 cells/mm^3^, according to current guidelines, could by itself have a substantial impact on HIV incidence, reducing the expected number of new HIV infections by 8.5% over 20 years. In comparison, initiation of treatment at diagnosis, the best-case scenario, resulted in a reduction of 11.7% over 20 years. The fact that the bulk of the benefit was associated with the intermediate case is not surprising considering that diagnosis, in the base case, occurs on average at CD4 300 cells/mm^3^, meaning that few individuals would be diagnosed in time to start ART at counts higher than 500 cells/mm^3^.

Viral load suppression on ART, another important variable in our test-and-treat strategy, required not only linkage, but also retention in care, as well as adherence to treatment. Although there is a fairly extensive literature on strategies to promote adherence to antiretroviral therapy [48]–[50], less is known about how to promote retention in care [51]–[53]. Meeting the intermediate targets for earlier initiation of treatment and viral load suppression may require an increased commitment to the provision of treatment and to programs that maintain individuals on treatment once they begin. Nearly 9,000 low-income individuals with HIV were on waiting lists to receive treatment in September 2011 [54], [55]. A longitudinal study in Nigeria showed that patients who started treatment at higher CD4 counts were at greater risk for dropping out of care and for non-adherence [56].

Implementing notification of test results and linkage to care to intermediate levels of improvement led to a more modest reduction in number of new HIV infections over 20 years (0.9% and 2.1% respectively). The reduced impact of these interventions seems to be due to already relatively high estimated notification and linkage rates and thus more limited room for improvement. If the proportion of persons tested with conventional testing is more than our assumed 50%, then improved notification may be more important. However, if the proportion of persons tested with rapid testing is more than our assumed 50%, then notification will be higher than our estimates; this may occur as rapid testing becomes more widely used.

The results of our sensitivity analyses indicated that a reduction in condom use to 50% from 75% among all urban infected MSM negated the benefits of the intervention. This finding underscores that safer sex practices such as condom use must be maintained in the MSM community.

Published reports vary regarding reductions in HIV transmission associated with suppression of plasma HIV RNA viral loads. By our estimate, even with an 80% reduction, a value that is at the lower bound of reported estimates, implementation of a test-and-treat strategy to intermediate levels of improvement could reduce the number of new infections by 26.4% over 20 years. If we assumed viral load suppression achieved a 99% reduction in the risk of HIV transmission, then the number of new infections over the same period was reduced by 51.6%. Our analyses conservatively assume that ART confers a reduction in transmission only when viral load suppression is achieved.

We also explored the ability of a test-and-treat strategy to achieve the shorter-term goals laid out by the National HIV/AIDS Strategy. We found that a combined test-and-treat strategy such as the one we have proposed that achieves intermediate levels of improvement in each intervention can meet four of the five goals of the National HIV/AIDS Strategy. Thus, a multi-component test-and-treat strategy could be a valuable part of the National HIV/AIDS Strategy.

Other models forecasting the effect of test-and-treat strategies on HIV epidemics in North America have been constructed and parameterized somewhat differently than ours. Our results are similar to those that assessed the impact of test-and-treat strategies on new HIV infections in British Columbia, where a 37% to 62% reduction in new cases over 25 years was estimated if the proportion of eligible individuals who received ART increased from 50% to 100% [7]. Another modeling exercise of the epidemic in the United States indicated that a strategy of test-and-treat could achieve an 18% reduction in new HIV infections over 20 years [10]. That model focused on low- and high-risk populations and assumed more modest reductions in risky sexual behaviors than we did (i.e., 20% vs. 50%) associated with the diagnosis of HIV infection.

Our model extrapolated past trends and current conditions into the future. If future conditions change with the emergence of more effective behavioral or biomedical prevention strategies, then the prevention benefit from the test-and-treat strategy we have proposed would likely change as well. Further, the benefits of a test-and-treat strategy as forecast by our model depend on correct estimates of levels of current implementation. For example, the timing of initiation of ART is an important parameter that we estimated using data from a cohort of HIV-infected MSM receiving care. However, this cohort may not be representative of all HIV-infected MSM, particularly those with limited access to care and persons who do not reside in metropolitan areas of the U.S. Similarly, our estimate that 80% of persons who initiate ART are able to achieve and maintain viral load suppression, likewise taken from HIV-infected MSM in care, may be optimistic. However, in both cases, our model likely underestimates the benefits of a test-and-treat strategy.

We did not assess the potential effect of antiretroviral resistance. Increases in the prevalence and transmission of antiretroviral resistant HIV could reduce population-based responsiveness to treatment that in turn reduces the effectiveness of a test-and-treat strategy. Findings from a previously published modeling exercise using population-level resistance data indicated that the effect of antiretroviral resistance on the projected number of new HIV cases was minimal [7].

Our analysis was based on New York City data. We assumed that the analysis may extend to other urban areas in the United States. However, for all estimates of current implementation, there is likely to be variation by race, ethnicity, age and geography.

The findings from our model provide decision makers with more information on how best to implement a test-and-treat strategy among MSM, highlighting the importance of a multipronged approach and allowing an assessment of which individual interventions might be most important to the success of such a strategy. It offers guidance based on reasonable implementation targets. It indicates how implementation of our multi-component test-and-treat strategy can attain the National HIV/AIDS Strategy goals. Ideally, findings from this model will spur more research on how best to improve implementation of the individual interventions; particularly interventions that increase the annual HIV testing rate and that improve adherence and retention in care. Our model suggests that a test-and-treat strategy could have a substantial impact on the urban MSM HIV epidemic, but would not replace the need for consistent condom by MSM.

## Supporting Information

Appendix S1 **Technical appendix to accompany “A Mathematical Model of Comprehensive Test-and-Treat Services and HIV Incidence among Men Who Have Sex with Men in the United States.”**
(DOCX)
