# Supplementary material for: A Mathematical Model of Comprehensive Test-and-Treat Services and HIV Incidence among Men Who Have Sex with Men in the United States
Source: PLoS One. 2012 Feb 10;7(2):e29098. doi: 10.1371/journal.pone.0029098 (PMC3277596; doi:10.1371/journal.pone.0029098)
Supplement: Appendix S1 — Technical appendix to accompany “A Mathematical Model of Comprehensive Test-and-Treat Services and HIV Incidence among Men Who Have Sex with Men in the United States.” (DOCX) [file pone.0029098.s001.docx]

**Appendix S1 to Accompany**

**A Mathematical Model of Comprehensive Test-and-Treat Services and HIV Incidence among Men Who Have Sex with Men in the United States**

Division of HIV/AIDS Prevention, National Center for HIV/AIDS, Viral Hepatitis, STD, and TB Prevention, Centers for Disease Control and Prevention, Atlanta, GA

**The findings and conclusions in this report are those of the authors and do not necessarily represent the official position of the Centers for Disease Control and Prevention.**

**Correspondence:**

Stephanie L. Sansom, Ph.D.

1600 Clifton Road, MS E-48

Atlanta, GA 30333

Phone: 404-639-6152

Fax: 404-639-8642

Email: sos97@cdc.gov

**Introduction**

We constructed a dynamic compartmental model of HIV infections among an urban population of MSM (based on New York City). Our model included only sexual transmission of HIV infection between MSM; it did not include MSM who are also injection drug users. Our model’s parameters for behavior, incidence and prevalence, and treatment were derived from CDC’s National HIV Behavioral Surveillance System (NHBS), the New York City HIV/AIDS Reporting System (HARS), and the CDC-sponsored HIV Outpatient Study (HOPS). NHBS is used to monitor prevalence and trends in HIV related risk behaviors, HIV testing and diagnosis, and the use of HIV prevention services among populations at high risk for acquiring HIV. In 2008, NHBS among MSM (NHBS-MSM2) collected data from 21 metropolitan statistical areas (MSAs) using an anonymous cross-sectional interview of men at locations and venues where MSM congregate. Respondents were offered anonymous HIV testing and diagnosis, regardless of self-reported HIV infection status [1]. The New York City HARS reports dates of HIV diagnosis and initial and subsequent CD4 cell counts for New York City residents who are diagnosed with HIV infection [2]. The HOPS is a prospective longitudinal cohort study of HIV-infected adults in care, many of whom are receiving antiretroviral treatment, seen at urban HIV specialty clinics in the United States. The HOPS includes data on HIV RNA viral load, CD4 cell count, and treatment regimens over time for each patient [3,4].

We divided the MSM population into compartments according to five infection stages, five age groups, and two sexual activity classes based on the number of partners an individual has in a year. The modeled infection stages included uninfected MSM who could become HIV-infected through anal intercourse based on the likelihood of forming a partnership with an infected person and the probability of HIV transmission within that partnership. Once infected, an individual progressed through the stages of acute infection, early latent infection, latent infection, late infection, and AIDS. At each infection stage, HIV-infected individuals could become aware of their infection through testing and diagnosis, and linked to care. HIV-infected individuals in care received or did not receive ART, with probability of ART initiation at a given level of CD4 cell count derived from estimates in the HOPS. The infection stages corresponded to CD4 cell counts: early latent infection corresponded to a CD4 count greater than 500 cells/mm^3^, latent infection to a CD4 count between 350 and 500 cells/mm^3^, late infection to a CD4 count between 200 and 350 cells/mm^3^, and AIDS to a CD4 count less than 200 cells/mm^3^ (Table 1).

We used per-contact transmission probabilities from a study of protected and unprotected insertive and receptive intercourse among MSM [5]. We modified those transmission probabilities to obtain per-contact transmission probabilities by each stage of infection using estimates in an observational study of treatment-naïve couples engaged in heterosexual intercourse [6]. We estimated the rate of transition from one stage of infection to another from that observational study.

Individuals in the various age groups (i.e., 18-24, 25-34, 35-44, 45-54, and 55-64 years) entered the sexually active population each year by aging, initiating sex with other men or immigration into the New York City geographic area. Individuals left the modeled population due to older age, HIV/AIDS-related death, or death from non-HIV/AIDS causes.

We assumed that individuals initiated ART according to published guidelines. Before 2009, initiation was generally recommended at CD4 < 350 cells/mm^3^. Since December 2009 treatment has been recommended at CD4 < 500 cells/mm^3^ and was recommended or considered optional at any CD4 cell count. [7] Survival estimates for individuals receiving ART depended on age and CD4 cell count at ART initiation [8,9].

Table 2 lists the symbols for key parameters used in our model and their values. We validated our model against epidemic data from the Department of Mental Health and Hygiene in New York City.

The effects of HIV testing and diagnosis and awareness of HIV positive status on reducing secondary HIV transmission were based in part on our assumption that individuals aware of their HIV infection decrease risky behaviors who could transmit HIV infection to their sexual partners. In our model, persons who were diagnosed with HIV infection decreased their number of sexual acts with uninfected partners by 15% [10]. Also they increased their use of condoms for anal sex from 50% to 75% [11-15]. We assumed a condom efficacy of 85% per sexual act [16-20] and varied assumptions about condom use in sensitivity analyses.

We evaluated the effect on the MSM HIV epidemic of a set of five interventions that could be included in a future test-and-treat strategy. Those interventions included increased HIV testing and diagnosis, improved notification of test results, improved linkage to care, earlier initiation of ART and more complete achievement of HIV viral load suppression. We established a current practice estimate for each intervention based on published data. Our model contains flows for the uninfected, HIV-infected but unaware of HIV status, HIV-infected and aware of HIV status after testing and diagnosis, persons linked to care after diagnosis, and persons linked to care and receiving ART (Figure 1).

This technical appendix describes the differential equations for the general model, per-contact transmission rates and per-partnership transmission probabilities, behavioral data and risk behaviors, and model calibration.

**System of differential equations for the general model**

The equations in our model use the following notation:

Six infection stages (h = 1,…,6) representing not HIV-infected (h = 1), acute (h = 2), early latent infection with CD4 count > 500 cells/mm^3^ (h = 3), latent infection with CD4 count between 350 cells/mm^3^ and 500 cells/mm^3^ (h=4), late infection with CD4 count between 200 cells/mm^3^ and 350 cells/mm^3^ (h = 5), and AIDS with CD4 < 200 cells/mm^3^ (h = 6) (Figure 1).

Transitions between infection stages occur by infection, progression from one HIV infection stage to another, aging, departure from the sexually active population, death from AIDS, not HIV-related death, and immigration into the sexually active population (at the not HIV-infected stage). The time a person spends in a specific infection stage h (where h > 1) before making a transition is assumed to be exponentially distributed with a mean 1 / (duration of stage h).

The five infection stages (h=2,3,4,5,6) are repeated four times for treatment status (r = 1, 2,3,4) representing populations unaware of their infection, (r=1); aware of their infection (r=2); aware of their infection and in-care (r=3); and in-care and on ART (r=4). Transition from treatment status r=1 to r=2 occurs because of testing and notification to the individual of test results. Transition from r=2 to r=3 occurs because of linkage to care. Transition from r=3 to r=4 occurs because of initiation of treatment by ART.

The treatment status for in-care and on ART (r=4) is handled differently from the others. In r=4 individuals do not transition from one infection stage to another. Instead they remain in a single stage where survival depends on age and their CD4 count when they began ART.

Let $X_{j,k}^{h,r}(t)$= the number of individuals in the population in HIV infection stage h, treatment status r, age group j (j = 1,…,5), and activity class k (k = 1,2) at time t.

For the not HIV-infected, h=1, the flows for all j, k are determined by

$\frac{dX_{j,k}^{h=1,r=1}(t)}{dt}=\Lambda_{j,k}-(\lambda_{j,k}^{r=1}(t)+\varepsilon_{j}+\mu_{j}-\delta_{j}^{+}+\delta_{j}^{-})X_{j,k}^{h=1,r=1}(t)$ (1)

Where

$\Lambda_{j,k}$= a constant in-immigration rate

$\varepsilon_{j}$ = exit rate from the sexually active population

$\mu_{j}$= the not HIV-related death rate

$\delta_{j}^{+}$and$\delta_{j}^{-}$= the aging rates into the age group and out of the age group respectively, and $\delta_{j}^{+}=\delta_{j-1}^{-}$

$\lambda_{j,k}^{r}(t)$ = the force of HIV infection, defined later.

Acute HIV infections (h=2) have similar equations for all j, k.

$\frac{dX_{j,k}^{h=2,r=1}(t)}{dt}=\lambda_{j,k}^{r=1}(t)X_{j,k}^{h=1,r=1}(t)-(\varepsilon_{j}+\mu_{j}-\delta_{j}^{+}+\delta_{j}^{-}+\tau_{j}^{h=2,r=1}+\omega^{h=2})X_{j,k}^{h=2,r=1}(t)$

$\frac{dX_{j,k}^{h=2,r=4}(t)}{dt}=\tau_{j}^{h=2,r=3}X_{j,k}^{h=2,r=3}(t)-(\varepsilon_{j}+\mu_{j}-\delta_{j}^{+}+\delta_{j}^{-}+\eta^{h=2,r=4}+\sigma_{j}^{h=2})X_{j,k}^{h=2,r=4}(t)$ (2)

Where

$\omega^{h}$= the rate of transition from infection stage h to stage h+1.

$\tau_{j}^{h,r}$= the rate of transition from treatment status r to status r+1 for r=1,2,3 and infection stage h and age j.

$\sigma_{j}^{h}$= death rate from ART for age j and infection stage h.

$\eta^{h,r}$= drop-out rate (1-retention rate) from in-care (r=3) or on ART (r=4).

= fraction linked to care in first year of HIV diagnosis.

Early latent and latent HIV infections (h=3 and 4) have similar equations for all j, k.

$\frac{dX_{j,k}^{h,r=1}(t)}{dt}=\omega^{h-1}X_{j,k}^{h-1,r=1}(t)-(\varepsilon_{j}+\mu_{j}-\delta_{j}^{+}+\delta_{j}^{-}+\tau_{j}^{h,r=1}+\omega^{h})X_{j,k}^{h,r=1}(t)$

$\frac{dX_{j,k}^{h,r=4}(t)}{dt}=\tau_{j}^{h,r=3}X_{j,k}^{h,r=3}(t)-(\varepsilon_{j}+\mu_{j}-\delta_{j}^{+}+\delta_{j}^{-}+\eta^{h,r=4}+\sigma_{j}^{h})X_{j,k}^{h,r=4}(t)$ (3)

Late HIV infections (h=5) have similar equations for all j, k.

$\frac{dX_{j,k}^{h=5,r=1}(t)}{dt}=\omega^{h=4}X_{j,k}^{h=4,r=1}(t)-(\varepsilon_{j}+\mu_{j}-\delta_{j}^{+}+\delta_{j}^{-}+\tau_{j}^{h=5,r=1}+\omega^{h=5})X_{j,k}^{h=5,r=1}(t)$

$\frac{dX_{j,k}^{h=5,r=4}(t)}{dt}=\tau_{j}^{h=5,r=3}X_{j,k}^{h=5,r=3}(t)-(\varepsilon_{j}+\mu_{j}-\delta_{j}^{+}+\delta_{j}^{-}+\eta^{h=5,r=4}+\sigma_{j}^{h=5})X_{j,k}^{h=5,r=4}(t)$ (4)

AIDS (h=6) after late HIV infections has similar equations for all j, k.

$\frac{dX_{j,k}^{h=6,r=1}(t)}{dt}=\omega^{h=5}X_{j,k}^{h=5,r=1}(t)-(\varepsilon_{j}+\mu_{j}-\delta_{j}^{+}+\delta_{j}^{-}+\tau_{j}^{h=6,r=1}+\Delta)X_{j,k}^{h=6,r=1}(t)$

$\frac{dX_{j,k}^{h=6,r=4}(t)}{dt}=\tau_{j}^{h=6,r=3}X_{j,k}^{h=6,r=3}(t)-(\varepsilon_{j}+\mu_{j}-\delta_{j}^{+}+\delta_{j}^{-}+\sigma_{j}^{h=6})X_{j,k}^{h=6,r=4}(t)$ (5)

Where

$\Delta$ = the death rate from AIDS.

The equations in the model are solved using Runge Kutta-4 algorithm with a time-step equal to one-fourth of a year.

**Per-contact transmission risks and per-partnership transmission probabilities**

We modeled per-partnership transmission probabilities based on the number of sex acts and per-act estimates of transmission probability according to a Bernoulli process, which assumes that contacts within a partnership are independent [21]. The per-contact transmission rates for MSM depend on infection stage – acute, latent, late, and AIDS – as well as circumcision status, condom usage, and type of sex act (e.g. insertive or receptive) [22-24]. We calculated the per-contact transmission rates for insertive and receptive anal intercourse between MSM based on overall per-contact transmission rates for MSM and per-contract transmission rates by infection stage for heterosexuals (Table 3). We adapted clinical trial data for heterosexual transmission to transmission among MSM. The second column in Table 3 shows an overall transmission rate per coital act for serodiscordant heterosexual couples, *Het(overall),* and the rates for acute infection, latent infection, late infection and AIDS, *Het(stage)* [6]. Next, we matched the overall transmission rates for heterosexual transmission against estimated transmission rates for insertive and receptive transmission among MSM from a prospective cohort study of HIV infection from specific acts [5]. The overall per-contact risk for unprotected receptive anal intercourse with an HIV infected partner, *URA_Infected_(overall),* is 0.0082, shown in the third column in Table 3. We calculated the remaining elements in the third column by multiplying the heterosexual transmission rate for each stage by the ratio of the overall transmission rate of unprotected receptive anal intercourse with an HIV infected partner to the overall heterosexual transmission rate.

$URA_{Infected}(stage)=Het(stage)\frac{URA_{Infected}(overall)}{Het(overall)}$ (10)

For example,

$$URA_{Infected}(AIDS)=(0.0043)\frac{0.0082}{0.0012}=0.0294$$

The cohort study estimated the overall per-contact risk for unprotected insertive anal intercourse with a partner of unknown infection status, *UIA_Unknown_(overall),* is 0.0006 and estimated the overall per-contact risk for unprotected receptive anal intercourse with a partner of unknown infection status, *URA_Unknown_(overall),* is 0.0027. We calculated the overall per-contact risk for unprotected insertive anal intercourse with an HIV infected partner, *UIA_Infected_(overall),* using the formula:

$$UIA_{Infected}(overall)=UIA_{Unknown}(overall)\frac{URA_{Infected}(overall)}{URA_{Unknown}(overall)}=(0.0006)\frac{0.0082}{0.0027}=0.0018$$

We show *UIA_Infected_(overall)* in the fourth column of Table 3.

We calculated the remaining elements in the fourth column, *UIA_Infected_(stage),* using the formula:

$UIA_{Infected}(stage)=Het(stage)\frac{UIA_{Infected}(overall)}{Het(overall)}$ (11)

For example,

$$UIA_{Infected}(AIDS)=(0.0043)\frac{0.0018}{0.0012}=0.0065$$

Per-contact transmission rates for persons on ART are not known. We assumed that persons who achieved viral load suppression experienced a 90% decline in the rate of per-contact HIV transmission compared with persons who do not achieve viral load suppression [25,26].

**Formulas for partnership transmission probability**

Approximately half of MSM had 3 or fewer partnerships per year and half had 4 or more partnerships per year. [1]. We calculated the median number of partnerships per age for each group using NHBS data (Table 4). The number of acts per partnership is unknown, but we assumed about 1.5 acts per week for each individual. This was in line with an estimate of 45.9 annual anal intercourse contacts per each partnership for 3 or fewer partners, and 3.55 annual anal contacts per each partnership for 4 or more partners [27].

The prevalence of male circumcision in the United States was estimated at 60%. Circumcision reduces the insertive infectivity for a not-HIV infected individual by 60%.

The transmission probability $\beta_{k}^{h,s}$ depends on HIV infection stage h for the partner, circumcision status s for the not HIV-infected, and activity class k. For$h=1$, $\beta_{k}^{h=1,s=1}=0$. The per-partnership transmission probability is given by the formula:

$$\beta_{k}^{h,s}=$$

$$1-(1-\Phi_{h})^{U_{k}\chi}(1-(1-c)\Phi_{h})^{P_{k}\chi}(1-(1-b_{s})\Gamma_{h})^{U_{k}(1-\chi)}(1-(1-c)(1-b_{s})\Gamma_{h})^{P_{k}(1-\chi)}$$

(12)

Where

*U_k_* = the number of unprotected contacts in a partnership with activity class k.

*P_k_* = the number of protected contacts in a partnership with activity class k.

$\Phi_{h}$and $\Gamma_{h}$= the per-contact transmission probability for receptive and insertive, unprotected anal intercourse respectively and infection stage h.

*c* = the condom effectiveness.

*b_s_* = the effectiveness from male circumcision (s = 1). b_s_ = 0 for uncircumcised (s = 2).

$\chi$ = the fraction of receptive contacts, so that (1 - $\chi$) is the fraction of insertive contacts.

**Formulas for the force of infection**

The formula for the force of HIV infection is a function of the number of sexual partners, the HIV transmission probability from infected partners in the different infection stages h, and the number of individuals in each age group, activity class, infection stage, and circumcision status. The probability of infection during the next partnership, assuming random mixing, for a not HIV-infected with circumcision status s is given by

$\varphi^{s}(t)=1-\mathop{\Pi}\limits_{h,j,k} (1-\beta_{k}^{h,s}(t))^{f_{j,k}^{h}(t)}$ (13)

Where

$f_{j,k}^{h}(t)=\frac{m_{j,k}\sum_{s} X_{j,k}^{h,s}(t)}{\sum_{j,k} m_{j,k}\sum_{h} \sum_{s} X_{j,k}^{h,s}(t)}$ (14)

$m_{j,k}$= the annual number of partners for persons of age j and activity class k.

The formula for $f_{j,k}^{h}(t)$ is the expected fraction of partnerships at time t for infection stage h, age group j, and activity class k.

The force of infection for Not HIV-infected with circumcision status s, age group j, and activity class k is given by

$\lambda_{j,k}^{s}(t)=1-(1-\varphi^{s}(t))^{m_{j,k}}$ (15)

**Additional information about behavioral data and risk behaviors**

We analyzed data collected during 2008 from the National HIV Behavioral Surveillance System among MSM (NHBS-MSM2) to estimate the number of partners per year and condom usage [1,28]. The National NHBS-MSM2 data set contains 8,153 participants from 21 metropolitan statistical areas. The New York City portion of NHBS contains 462 participants. We compared our estimates from the national sample and from the New York City subset and found very similar results in aggregate. When we divided the data into smaller strata by age and activity class, the national sample gave more precise estimates.

The participants may be more sexually active than the general MSM population and may not represent all MSM. However recent reports suggest that venue-based sampling schemes provide reasonable estimates of MSM populations in urban areas [29-31].

**New York City population data and model calibration**

We calibrated the dynamic compartment model to population data from New York City in 2004. From the New York City Community Health Survey [32], we estimated the population of MSM in New York City in 2004 at 194,000. For our model we assumed that the MSM population size has been constant but would have obtained similar results by assuming small growth or decline.

The Community Health Survey gave us estimates of the population by age group for MSM in 2004 (Table 5). The estimates for age group 18-24 and 55-64 were based on small sample sizes and may undercount those groups relative to other age groups.

The calibration for our model was a three-step process. First, we determined an initial population in 1975 and a constant annual input for each age group of uninfected MSM (Table 6) so that the model’s calculations matched the age group’s population in 2004). We began with the first age group (18-24), and then we determined the second age group (25-34), and so on.

The second step in calibration was to determine the rate that persons exited from sexual activity. Everyone aged 65 and over exited the model. In addition we set an exit rate of 2.6% from age group 4 (45-54) so that we equaled the size of that age group in New York City data in 2004. We also set an exit rate of 36.0% from age group 5 (55-64) to equal the size of that age group in New York City data in 2004.

The third step in calibration was to fit a least squares curve to reported diagnoses in AIDS from 1996-2008 based on surveillance data from New York City (Figure 2) [33]. A percentage of the population by age and activity was assumed to be already infected with HIV at the start of the simulation. The percentage was selected to provide the best least squares fit.

As a result of the calibration, the 2004 value within the model for the population of MSM in New York City is 193,737 (compared to 194.000 from the NYC Community Health Survey).

**Internal and external validation of the model**

We compared our model’s results to New York City data (Table 5). Population data by age group and overall reported diagnoses in AIDS were used for calibration as described above.

AIDS diagnoses by age group, average age for AIDS diagnoses, range of average age for AIDS diagnoses, and prevalence of HIV/AIDS in the MSM population were not used in calibration and provide an external validation of our model. The average age of reported AIDS diagnosis in our model is about 2 years higher than in New York City surveillance data. In particular our model shows fewer AIDS diagnoses in the 35-44 age group and more AIDS diagnoses in the 45-54 age group.

Our model calculated prevalence in 2010 at 15.0%. This prevalence estimate is between 13.7% (95% C.I. 6.0%-28.3%) based on 55 MSM from the New York City Health and Nutrition Examination Survey 2004[34], and 29% (95% C.I. 25%-33%) for New York City based on 462 MSM in NHBS-MSM2 2008 [1].

We estimated the diagnostic rate by age and HIV infection stage by comparing the fraction aware by age in our model with the fraction aware by age from NHBS-MSM2 (Figure 3).

**Table 1. Infection stage and parameter values.**

| Infection Stage | CD4 Count Range, Cells/mm^3^ | Heterosexual Per-Contact Transmission Probability | Duration In Infection Stage |
| --- | --- | --- | --- |
| Acute |  | 0.0082 | 4 months |
| Early Latent (Asymptomatic) | > 500 | 0.0007 | 3.5 years |
| Latent (Asymptomatic) | 350 - 500 | 0.0007 | 3.5 years |
| Late (Symptomatic) | 200 - 350 | 0.0014 | 2 years |
| AIDS | < 200 | 0.0043 | 1 year |

**Table 2. Symbols and values for input parameters.**

| Symbol | Definition | Values |
| --- | --- | --- |
| $X_{j,k}^{h,r}(t)$ | Number of individuals in the population in HIV infection stage h, treatment status r, age group j (j = 1,…,5), and activity class k (k = 1,2) at time t. | Calculated dynamically in the model. |
| $\Lambda_{j,k}$ | Constant in-immigration rate. | 0.16 (j=1); 0.067 (j=2); 0.045 (j=3); 0.03 (j=4); 0 (j=5) |
| $\varepsilon_{j}$ | Exit rate from the sexually active population. | 0 (j=1,2,3): 0.025 (j=4); 0.33 (j=5) |
| $\mu_{j}$ | Not HIV-related death rate. | 0.001264 (j=1); 0.001352 (j=2); 0.00242 (j=3); 0.005657 (j=4); 0.11858 (j=5) |
| $\delta_{j}^{+}$and$\delta_{j}^{-}$ | Aging rates into the age group and out of the age group respectively, and $\delta_{j}^{+}=\delta_{j-1}^{-}$ . | 1/7 (j=1); 1/10 (j=2,3,4,5) |
| $\lambda_{j,k}^{r}(t)$ | Force of HIV infection, defined later. | Calculated dynamically in the model. |
| $\omega^{h}$ | Rate of transition from infection stage h to stage h+1. | 1/duration h where duration = 0.2 (h=2); 3.5 (h=3,4); 2 (h=5) |
| $\tau^{h,r}_{j}$ | Rate of transition from treatment status r to status r+1 for r=1,2,3 and infection stage h and age j. | Testing varies from 0.05 (h=2, j=1) to 1 (h=6, j=5).  Linkage to care (r=2) 0.7 in first year; 0.1 in subsequent years).  Initiation of ART (r=3) varies from 0 (h=2) to 0.606 (h=6). |
| $\sigma_{j}^{h}$ | Death rate from ART for age j and infection stage h. | Varies from 0.00359 (h=2, j=1) to 0,04113 (h=6, j=5). |
| $\eta^{h,r}$ | Drop-out rate (1-retention rate) from in-care (r=3) or on ART (r=4). | 0.15 (r=3, all h); 0.2 do not achieve VL suppression (r=4, all h). |
|  | Fraction linked to care in first year of HIV diagnosis. | .7 |
| $\Delta$ | Death rate from AIDS. | 1/duration h=6 where duration h=6 is 1.5 |
| $\beta_{k}^{h,s}$ | Transmission probability depends on HIV infection. | Calculated based on other parameters. |
| *U_k_* | Number of unprotected contacts in a partnership with activity class k. | 74.3x0.5 = 37.1 (k=1)  5.78x0.5 = 2.89 (k=2) |
| *P_k_* | Number of protected contacts in a partnership with activity class k. | 37.1 (k=1)  2.89 (k=2) |
| $\Phi_{h}$and $\Gamma_{h}$ | Per-contact transmission probability for receptive and insertive, unprotected anal intercourse respectively and infection stage h. | Calculated in the model from other parameters. |
| *c* | Condom effectiveness. | 0.85 |
| *b_s_* | Effectiveness from male circumcision. | 0 for receptive, 0.6 for insertive (s=1); 0 for uncircumcised (s=2). |
| $\chi$ | Fraction of receptive contacts, so that (1 - $\chi$) is the fraction of insertive contacts. | 0.5 |
|  | Probability of infection during the next partnership, assuming random mixing, for a not HIV-infected with circumcision status s. | Calculated dynamically in the model. |
| $m_{j,k}$ | Annual number of partners for persons of age j and activity class k. | Varies from 1.61 to 1.89 (k=1) and from 13.91 to 25.7 (k=2). |
| $f_{j,k}^{h}(t)$ | Expected fraction of partnerships at time t for infection stage h, age group j, and activity class k. | Calculated dynamically in the model. |

**Table 3. Per-contact transmission rates for serodiscordant heterosexual and MSM couples.**

| Infection Stage | Heterosexual, Het(stage) | MSM, Unprotected Receptive Anal Intercourse with HIV Infected Partner, *URA_Infected_(stage)* | MSM, Unprotected Insertive Anal Intercourse with HIV Infected Partner, *UIA_Infected_(stage)* |
| --- | --- | --- | --- |
| Overall | 0.0012 | 0.0082 | 0.0018 |
| Acute | 0.0082 | 0.0560 | 0.0123 |
| Latent (Asymptomatic) | 0.0007 | 0.0048 | 0.0011 |
| Late (Symptomatic) | 0.0014 | 0.0096 | 0.0021 |
| AIDS | 0.0043 | 0.0294 | 0.0065 |

**Table 4. Median partnerships per year by age and activity level.**

| Age | Partnerships for Lower Sexual Activity Class | Partnerships for Higher Sexual Activity Class |
| --- | --- | --- |
| 18-24 | 1.89 | 13.91 |
| 25-34 | 1.76 | 20.62 |
| 35-44 | 1.70 | 24.80 |
| 45-54 | 1.66 | 22.46 |
| 55-64 | 1.61 | 25.70 |

**Table 5. Comparing our model to New York City data, 2004.**

| Variable | Calibrated To NYC Data | Model Estimate: 2004 | New York City Data: 2004 | Source For New York City Data |
| --- | --- | --- | --- | --- |
| Population of MSM: Age 18-24 | Yes | 24,657 | 24,635 | NYC Community Health Survey, 2004 |
| Population of MSM: Age 25-34 | Yes | 55,910 | 55,429 | NYC Community Health Survey, 2004 |
| Population of MSM: Age 35-44 | Yes | 59,857 | 60,561 | NYC Community Health Survey, 2004 |
| Population of MSM: Age 45-54 | Yes | 44,063 | 44,138 | NYC Community Health Survey, 2004 |
| Population of MSM: Age 55-64 | Yes | 9,249 | 9,239 | NYC Community Health Survey, 2004 |
| Total Population of MSM: Age 18-64 | No | 193,737 | 194,000 | NYC Community Health Survey, 2004 |
| Reported AIDS Diagnoses: Age 18-24 | No | 67 | 81 | NYC HARS, 2004 |
| Reported AIDS Diagnoses: Age 25-34 | No | 294 | 311 | NYC HARS, 2004 |
| Reported AIDS Diagnoses: Age 35-44 | No | 413 | 478 | NYC HARS, 2004 |
| Reported AIDS Diagnoses: Age 45-54 | No | 329 | 199 | NYC HARS, 2004 |
| Reported AIDS Diagnoses: Age 55-64 | No | 70 | 63 | NYC HARS, 2004 |
| Total Reported AIDS Diagnoses: Age 18-64 | Yes | 1,173 | 1,150 | NYC HARS, 2004 |

**Table 6. Calculated initial values for population in 1975 and constant annual input.**

| Variable | Lower Sexual Activity Class | Higher Sexual Activity Class |
| --- | --- | --- |
| Initial Population of MSM, 1975 |  |  |
| Age 18-24, h=1 | 12,319 | 12,319 |
| Age 25-34, h=1 | 27,742 | 27,742 |
| Age 35-44, h=1 | 30,264 | 30,264 |
| Age 45-54, h=1 | 22,116 | 22,116 |
| Age 55-64, h=1 | 4,656 | 4,656 |
| Age 18-24, h=2 | 7.3914 | 7.3914 |
| Age 25-34, h=2 | 16.6542 | 16.6542 |
| Age 35-44, h=2 | 18.1584 | 18.1584 |
| Age 45-54, h=2 | 13.2696 | 13.2696 |
| Age 55-64, h=2 | 2.7936 | 2.7936 |
| Age 18-24, h=3+4 total | 7.3914 | 7.3914 |
| Age 25-34, h=3+4 total | 16.6542 | 16.6542 |
| Age 35-44, h=3+4 total | 18.1584 | 18.1584 |
| Age 45-54, h=3+4 total | 13.2696 | 13.2696 |
| Age 55-64, h=3+4 total | 2.7936 | 2.7936 |
| Constant Annual Input |  |  |
| Age 18-24, h=1 | 1,971.04 | 1,971.04 |
| Age 25-34, h=1 | 1,858.714 | 1,858.714 |
| Age 35-44, h=1 | 1,361.88 | 1,361.88 |
| Age 45-54, h=1 | 663.48 | 663.48 |
| Age 55-64, h=1 | 0 | 0 |

**Figure 1. Compartmental model of HIV infection and disease progression.**

**Figure 2. Validating our model against new AIDS diagnoses in New York City, 1997-2008 – MSM.**

**Figure 3. Estimated annual testing rate of 24% for HIV-infected MSM matches the fraction who are aware by age, 2008.**

**References**

**1. Centers for Disease Control and Prevention (2010) Prevalence and awareness of HIV infection among men who have sex with men--21 cities, United States, 2008. MMWR Morb Mortal Wkly Rep 59: 1201-1207.**

**2. Torian LV, Wiewel EW, Liu K-L, Sackoff JE, Frieden TR (2008) Risk factors for delayed initiation of medical care after diagnosis of human immunodeficiency virus. Arch of Intern Med 168: 1181-1187.**

**3. Buchacz K, Baker R, Palella F, Chmiel J, Lichtenstein K, et al. (2010) AIDS-defining opportunistic illnesses in US patients, 1994-2007: a cohort study. AIDS 24: 1549-1559.**

**4. Palella FJ, Delaney KM, Moorman AC, Loveless MO, Fuhrer J, et al. (1998) Declining morbidity and mortality among patients with advanced human immunodeficiency virus infection. New Engl J Med 338: 853-860.**

**5. Vittinghoff E, Douglas J, Judon F, McKiman D, MacQueen K, et al. (1999) Per-contact risk of human immunodificiency virus tramnsmision between male sexual partners. Am J Epidemiol 150: 306-311.**

**6. Wawer Maria J, Gray Ronald H, Sewankambo Nelson K, Serwadda D, Li X, et al. (2005) Rates of HIV-1 transmission per coital act, by stage of HIV-1 infection, in Rakai, Uganda. J Infect Dis 191: 1403-1409.**

**7. DHHS Panel on Antiretroviral Guidelines for Adults and Adolescents. Guidelines for the Use of Antiretroviral Agents in HIV-1-Infected Adults and Adolescents. Original document published November 2008. Version of January 2011.** [**http://www.aidsinfo.nih.gov/Guidelines/**](http://www.aidsinfo.nih.gov/Guidelines/)**. Accessed April 28, 2011.**

**8. May M, Sterne JAC, Sabin C, Costagliola D, Justice A, et al. (2007) Prognosis of HIV-1-infected patients up to 5 years after initiation of HAART: collaborative analysis of prospective studies. AIDS 21: 1185-1197.**

**9. The Antiretroviral Therapy Cohort Collaboration (2008) Life expectancy of individuals on combination antiretroviral therapy in high-income countries: a collaborative analysis of 14 cohort studies. Lancet 372: 293-299.**

**10. Marks G, Millett G, Bingham T, Lauby J, Murrill C, et al. (2010) Prevalence and protective value of serosorting and strategic positioning among Black and Latino men who have sex with men. Sex Transm Dis 37: 325-327.**

**11. Crepaz N, Marks G, Liau A, Mullins M, Aupont L, et al. (2009) Prevalence of unprotected anal intercourse among HIV-diagnosed MSM in the United States: a meta-analysis. AIDS 23: 1617-1629.**

**12. Marks G, Crepaz N (2001) HIV-positive men's sexual practices in the context of self-disclosure of HIV status. J Acquir Immune Defic Syndr 27: 79-85.**

**13. Marks G, Crepaz N, Janssen R (2006) Estimating sexual transmission of HIV from persons aware and unaware that they are infected with the virus in the USA. AIDS 20: 1447-1450.**

**14. Marks G, Crepaz N, Senterfitt JW, Janssen R (2005) Meta-analysis of high-risk sexual behavior in persons aware and unaware they are infected with HIV in the United States: implications for HIV prevention programs. J Acquir Immune Defic Syndr 39: 446-453.**

**15. Marks G, Millett G, Bingham T, Bond L, Lauby J, et al. (2009) Understanding differences in HIV sexual transmission among Latino and black men who have sex with men: The Brothers y Hermanos Study. AIDS Behav 13: 682-690.**

**16. Crosby R, DiClemente RJ, Holtgrave DR, Wingood GM (2002) Design, measurement, and analytical considerations for testing hypotheses relative to condom effectiveness against non-viral STIs. Sex Transm Infect 78: 228-231.**

**17. Davis KR, Weller SC (1999) The effectiveness of condoms in reducing heterosexual transmission of HIV. Fam Plann Perspect 31: 272-279.**

**18. Weller SC, Davis-Beaty K. Condom effectiveness in reducing heterosexual HIV transmission (review). *Cochrane Database Syst Rev 2002.* (1, Art. No.: CD003255. doi:10.1002/14651858).** [**http://www.thechochranelibrary.com**](http://www.thechochranelibrary.com)**.**

**19. Foss A, Watts C, Vickerman P, Heise L (2004) Condoms and prevention of HIV. BMJ 329: 185-186.**

**20. Giesecke J, Scalia-Tomba G, Gthberg M, Tull P (1992) Sexual behaviour related to the spread of sexually transmitted diseases--a population-based survey. Int J STD AIDS 3: 255-260.**

**21. Pinkerton S, Abramson P (1998) The Bernoulli-process model of HIV transmission: applications and implications. In: Holtgrave D, editor. Handbook of Economic Evaluation of HIV Prevention Programs. New York: Plenum Press.**

**22. Baggaley R, White R, Boily M-C (2010) HIV transmission risk through anal intercourse: systematic review, meta-analysis and implications for HIV prevention. Int J Epidemiol 39: 1048-1063.**

**23. Leynaert B, Downs AM, de Vincenzi I, European Study Group on Heterosexual Transmission of HIV (1998) Heterosexual transmission of human immunodeficiency virus: variability of infectivity throughout the course of infection. Am J Epidemiol 148: 88-96.**

**24. Osmond D, Bacchetti P, Chaisson RE, Kelly T, Stempel R, et al. (1988) Time of exposure and risk of HIV infection in homosexual partners of men with AIDS. Am J Public Health 78: 944-948.**

**25. Porco TC, Martin JN, Page-Shafer KA, Cheng A, Charlebois E, et al. (2004) Decline in HIV infectivity following the introduction of highly active antiretroviral therapy. AIDS 18: 81-88.**

**26. Attia S, Egger M, Muller M, Zwahlen M, Low N (2009) Sexual transmission of HIV according to viral load and antiretroviral therapy: systematic review and meta-analysis. AIDS 23: 1397-1404.**

**27. The Healthy Living Project Team (2007) Effects of a behavioral intervention to reduce risk of transmission among people living with HIV: The Healthy Living Project Randomized Controlled Study. JAIDS 44: 213-221.**

**28. MacKellar D, Gallagher K, Finlayson T, Sanchez T, Lansky A, et al. (2007) Surveillance of HIV risk and prevention behaviors of men who have sex with men--a national application of venue-based, time-space sampling. Public Health Rep 122 Suppl 1: 39-47.**

**29. Xia Q, Tholandi M, Osmond DH, Pollack LM, Zhou W, et al. (2006) The effect of venue sampling on estimates of HIV prevalence and sexual risk behaviors in men who have sex with men. Sex Transm Dis 33: 545-550.**

**30. Pollack LM, Osmond DH, Paul JP, Catania JA (2005) Evaluation of the Center for Disease Control and Prevention's HIV Behavioral Surveillance of Men Who Have Sex With Men: sampling issues. Sex Transm Dis 32: 581-589.**

**31. Pollack LM, Osmond DH, Paul JP, Catania JA (2006) Letter to the Editor. Sex Transm Dis 33: 274-275.**

**32. New York City Department of Health and Mental Hygiene. Community Health Survey.** [**http://nyc.gov/html/doh/html/survey/survey.html**](http://nyc.gov/html/doh/html/survey/survey.html)**. Accessed March 17, 2010.**

**33. New York City Department of Health and Mental Hygiene. New York City HIV/AIDS annual surveillance statistics 2009. 2010;** [**http://www.nyc.gov/html/doh/html/ah/hivtables.shtml**](http://www.nyc.gov/html/doh/html/ah/hivtables.shtml)**. Accessed April 28, 2011.**

**34. Nguyen TQ, Gwynn RC, Kellerman SE, Begier E, Garg RK, et al. (2008) Population prevalence of reported and unreported HIV and related behaviors among the household adult population in New York City, 2004. AIDS 22: 281-287.**
